# Exosomes secreted by chronic hepatitis B patients with PNALT and liver inflammation grade ≥ A2 promoted the progression of liver cancer by transferring miR‐25‐3p to inhibit the co‐expression of TCF21 and HHIP

**DOI:** 10.1111/cpr.12833

**Published:** 2020-06-11

**Authors:** Yi Ouyang, Yujing Tang, Lei Fu, Shifang Peng, Wanfeng Wu, Deming Tan, Xiaoyu Fu

**Affiliations:** ^1^ Key Laboratory of Viral Hepatitis Department of Infectious Diseases Xiangya Hospital Central South University Changsha China; ^2^ Department of Second Area of Liver Disease Xia'men Hospital of Chinese Medicine Xia'men China; ^3^ School of the Integrated Traditional Chinese and Western Medicine Hunan University of Chinese Medicine Changsha China

**Keywords:** CHB, exosome, HHIP, liver cancer, miR‐25‐3p, TCF21

## Abstract

**Objectives:**

The current study aimed to investigate the mechanism by which exosomes secreted by CHB patients with PNALT and liver inflammation grade (≥A2) affected the development of liver cancer.

**Materials and methods:**

Gene expression was assessed by RT‐PCR, Western blotting and immunohistochemistry. CCK‐8, colony formation, transwell, scratch‐wound and flow cytometry assays were used to detect cell viability, proliferation, apoptosis and metastasis. The interaction of TCF21 and HHIP was assessed by co‐immunoprecipitation assay. Luciferase reporter was used to detect the combination of TCF21/HHIP and miR‐25‐3p. Xenograft studies in nude mice manifested tumour growth ability of miR‐25‐3p. Bioinformatics analyses were conducted using TargetScan, EVmiRNA, TCGA, GEO, DAVID**,** COEXPEDIA**,** UALCAN, UCSC and the Human Protein Atlas databases**.**

**Results:**

CHB‐PNALT‐Exo (≥A2) promoted the proliferation and metastasis of HepG2.2.15 cells. miR‐25‐3p was upregulated in CHB‐PNALT‐Exo (≥A2). miR‐25‐3p overexpression promoted cell proliferation and metastasis and was related to poor survival in patients with CHB‐PNALT (≥A2). The cell proliferation‐ and metastasis‐promoting functions of CHB‐PNALT‐Exo (≥A2) were abolished by miR‐25‐3p inhibitors. TCF21 directly interacted with HHIP. Inhibition of TCF21 or HHIP promoted cell proliferation and metastasis. Knockdown of TCF21 or HHIP counteracted the effects of CHB‐PNALT‐Exo (≥A2) containing miR‐25‐3p inhibitor on cell proliferation, metastasis and the expression of Ki67, E‐cadherin and caspase‐3/‐9.

**Conclusions:**

Transfer of miR‐25‐3p by CHB‐PNALT‐Exo promoted the development of liver cancer by inhibiting the co‐expression of TCF21 and HHIP.

## 1 INTRODUCTION

Primary liver cancer ranks sixth among all the malignant cancers in terms of its morbidity and is the second most common cause of cancer‐related deaths, with about 700 000 deaths annually worldwide.^1^, ^2^ In China, primary hepatic cancer is the second most common cancer after liver cancer.^3^ Hepatic cells secret extracellular vesicles (30 nm‐150 nm in diameter) called exosomes that contain numerous DNAs, microRNAs (miRNAs), mRNAs, proteins and lipids.^4^ Exosomes have been shown to modulate the exchange of these substances between cells and play a vital role in maintaining hepatic homeostasis. Exosomes secreted at different developmental stages or induced by various stimuli differ, and they play important roles in physiological, cellular and pathological processes.^5^, ^6^ Exosomes have been shown to influence hepatic cell proliferation and inflammation.^7^ Statistics show that more than 80% of the primary hepatic cancer patients are HBV HBsAg‐positive,^8^ and liver cirrhosis caused by chronic HBV infection is a major risk factor for hepatocellular carcinoma (HCC). Alanine aminotransferase (ALT) is the most common biochemical indicator used to evaluate liver inflammation,^9^ and current guidelines for the prevention and treatment of hepatitis B^10^ suggested that chronic hepatitis B (CHB) patients with persistently normal alanine aminotransferase levels (PNALT), and the infection may develop into liver cirrhosis or cancer. The risk of HCC in patients with chronic HBV infection has been estimated to be 100 times than that of non‐infected patients.^11^ However, the risk of HCC in CHB patients, with ALT levels more than twice the ULN, is higher than that of patients with PNALT. Therefore, the related molecular mechanisms need to be further explored.

MicroRNA (miRNA) is a type of short (~22 nucleotides in length), non‐coding single‐stranded RNA. Most miRNAs are enriched in exosomes,^12^ which are small vesicles (30 nm‐100 nm in diameter) containing a large number of miRNAs, mRNAs and proteins. The pool of exosomal miRNAs is more homogeneous and more stable than the pool of free miRNAs. Exosome‐derived miRNAs have been shown to have numerous functions, which has been reported that overexpression of miR‐18a in exosomes promoted the proliferation of liver cells and decreased the levels of α‐estrogen receptor.^13^ Additionally, exosomes derived from HBV‐associated liver cancer promoted chemoresistance by upregulating chaperone‐mediated autophagy.^14^ Exosomal miRNAs derived from different cells have distinct effects in various diseases. For example, mesenchymal stem cell–derived exosomal microRNA‐133b suppresses the progression of glioma.^12^ Exosome‐mediated transfer of miR‐133b from multipotent mesenchymal stromal cells to neural cells contributes to neurite outgrowth.^15^ Additionally, disease‐derived exosomal miRNAs have been shown to have distinct effects. For example, it has been reported that cancer‐derived exosomal miR‐25‐3p promotes the formation of a pre‐metastatic niche formation by inducing vascular permeability and angiogenesis,^16^ and platelet‐derived exosomal miRNA‐25‐3p inhibits coronary vascular endothelial cell inflammation in ApoE^−/−^ mice.^17^ Therefore, we hypothesized that exosomal miRNA‐25‐3p might play a vital role in the genesis and progression of liver cancer.

## 2 MATERIALS AND METHODS

### 2.1 Samples collection

Whole blood samples collected from 48 CHB patients with PNALT and liver inflammation grade ≥ A2 and 15 CHB patients with PNALT and liver inflammation grade < A2, as well as tumour and adjacent normal tissue samples collected from 48 HBV‐positive patients with liver cancer resected during surgical procedures, were obtained from the Xiangya Hospital Affiliated to Central South University from January 2016 to December 2018. The clinical characteristic of the study patients has been listed in Tables 1 and 2. Collected tissues were stored in liquid nitrogen. Liver inflammation grade was evaluated by two pathologists according to the METAVIR grading system. Each participant provided written informed consent. The use of human clinical tissues was approved by the Institutional Human Experiment and Ethics Committee of Xiangya Hospital.

**TABLE 1:** Clinical characteristics of CHB‐PNALT of patients

Group	ID	Age	Gender	ALT/(U L^−1^)	AST/(U L^−1^)	HBeAg	HBV‐DNA/(U mL^−1^)	Liver tissue inflammation grade
≥A2	1	34	Male	37.9	17.4	+	6.92E + 06	A2
	2	31	Male	38.6	14	+	9.06E + 07	A2
	3	23	Male	28.2	11.8	+	3.48E + 07	A2
	4	28	Male	32.9	20.1	+	8.19E + 07	A3
	5	26	Male	30.5	22.8	+	4.11E + 07	A2
	6	29	Male	33.8	16.2	+	1.97E + 08	A3
	7	36	Male	32.6	18.7	+	2.93E + 08	A3
	8	27	Male	36.7	21.3	+	3.65E + 07	A3
	9	32	Male	28.9	10.7	+	8.32E + 07	A2
	10	34	Male	35.3	12.5	+	7.37E + 07	A2
	11	30	Male	30.1	15.1	+	5.83E + 08	A3
	12	31	Male	29.7	12.3	+	6.23E + 07	A3
	13	32	Male	31.4	14.6	+	1.22E + 06	A2
	14	25	Male	30.5	11.5	+	4.25E + 06	A2
	15	20	Male	28.9	18.2	+	5.84E + 07	A2
	16	22	Male	32.3	20.1	+	2.33E + 08	A3
	17	32	Male	37.9	21.6	+	8.05E + 08	A2
	18	36	Male	34.5	15.6	+	7.11E + 07	A2
	19	38	Male	36.4	18.7	+	5.42E + 07	A3
	20	40	Male	35.2	19.5	+	3.37E + 06	A3
	21	21	Male	29.7	14.2	+	2.36E + 07	A2
	22	25	Male	30.5	16.5	+	6.14E + 07	A2
	23	26	Male	30.8	15.2	+	4.16E + 08	A3
	24	30	Male	31.2	15.5	+	3.84E + 08	A3
	25	33	Male	31.6	14.7	+	4.26E + 07	A2
	26	32	Male	32.5	18.2	+	7.29E + 07	A2
	27	35	Male	37.5	19.1	+	3.86E + 07	A2
	28	36	Male	36.5	14.5	+	2.89E + 08	A3
	29	30	Male	35.6	17.5	+	5.03E + 07	A2
	30	31	Male	38.1	18.6	+	3.71E + 06	A2
	31	25	Male	29.4	13.5	+	2.64E + 06	A2
	32	28	Male	28.9	16.5	+	2.08E + 08	A3
	33	30	Male	26.7	15.7	+	6.16E + 08	A2
	34	29	Male	24.3	18.4	+	5.32E + 07	A2
	35	34	Male	23.1	19.1	+	4.98E + 07	A3
	36	25	Male	22.6	20.2	+	4.05E + 07	A3
	37	26	Male	30.8	21.3	+	6.74E + 08	A2
	38	28	Male	36.4	18.9	+	4.21E + 07	A3
	39	32	Male	37.8	16	+	3.64E + 06	A2
	40	33	Male	36.5	17.8	+	2.84E + 06	A2
	41	35	Male	35.4	16.2	+	1.84E + 07	A2
	42	36	Male	31.2	17.5	+	2.44E + 08	A3
	43	37	Male	32.5	18.1	+	5.09E + 08	A2
	44	35	Male	33.5	16.7	+	3.65E + 07	A2
	45	31	Male	34.4	18.2	+	3.58E + 07	A3
	46	21	Male	35.7	16.1	+	5.74E + 08	A2
	47	20	Male	25.6	18.9	+	5.73E + 08	A3
	48	26	Male	23.6	16.2	+	3.15E + 07	A2
<A2	1	29	Male	33.8	16.2	+	1.97E + 08	A1
	2	36	Male	32.6	18.7	+	2.93E + 08	A1
	3	27	Male	36.7	21.3	+	3.65E + 07	A1
	4	32	Male	28.9	10.7	+	8.32E + 07	A1
	5	34	Male	35.3	12.5	+	7.37E + 06	A1
	6	22	Male	31.5	11.5	+	2.88E + 08	A1
	7	26	Male	30.8	20.8	+	3.14E + 08	A1
	8	28	Male	29.6	16.7	+	3.05E + 07	A1
	9	30	Male	30.5	11.6	+	7.62E + 07	A1
	10	31	Male	31.9	19.4	+	8.13E + 06	A1
	11	33	Male	32.6	15.2	+	3.08E + 08	A1
	12	33	Male	35.5	18.7	+	4.56E + 08	A1
	13	35	Male	34.1	15.3	+	5.77E + 07	A1
	14	36	Male	33.8	12.9	+	6.89E + 07	A1
	15	38	Male	28.9	11.4	+	7.14E + 06	A1

**TABLE 2:** Correlation of miR‐25‐3p expression with clinicopathological parameters

Clinical feature	No of patients (n = 48)	miR‐25‐3p expression		*P*‐value
		Low (n = 30)	High (n = 18)	
Gender				
Female	28	18	10	.964
Male	20	12	8	
Age (y)				
≥50	23	15	8	.224
<50	25	15	10	
AFP (ng/mL)				
≤400	22	13	9	.366
>400	26	17	9	
Tumour size (cm)				
≤5	23	21	2	.000**
>5	25	9	16	
Tumour number				
Single	27	23	4	.039**
Multiple	21	7	14	
Tumour differentiation				
I‐II	26	21	5	.000**
III‐IV	22	9	15	
Vascular invasion				
No	20	18	2	.000**
Yes	28	12	16	
TNM stage				
I	19	18	1	.000**
II	14	7	7	
III	15	5	10	

Note

Chi‐square test.

Abbreviations: AFP, alpha‐fetoprotein; TNM, tumour‐node‐metastasis.

** *P* < .05.

### 2.2 Cell culture

HepG2.2.15 (HBV‐positive liver cancer cell line) was purchased from the Institutes for Biological Sciences at the Chinese Academy of Sciences (Shanghai, China). All cells were cultured in high‐glucose Dulbecco's modified Eagle's medium (DMEM) supplemented with 10% foetal bovine serum (FBS; Thermo Fisher Scientific, Inc). All the cells were cultured in a humidified incubator with 5% CO_2_ at 37°C.

### 2.3 Isolation and identification of exosomes

Exosomes were isolated according to Exosome Isolation Reagent protocols (GS™ Exosome Isolation Reagent, Geneseed Biotech). Exosomes vesicles were resuspended in 100‐200 µL PBS and stored at −80°C for further use. TSG101 (the biomarkers of exosomes) was identified using Western blotting, and the protein in free serum was used as a positive control. The preparations were examined, and images were captured using transmission electron microscopy (TEM; JEM‐2100; JEOL, Ltd.). Each isolation was verified by nanoparticle tracking analysis using a Nanosight NS300 (Nanosight Ltd.) to determine the size and quantity of EVs extracted. Then, exosomes (10 μL) were added into PBS (1000 μL) and exosomes' diameter was tested using Zetasizer Nano Series—Nano‐ZS.

### 2.4 Exosome uptake

Purified exosomes from CHB patients with PNALT (CHB‐PNALT‐Exo) were labelled with 1 μmol/L Dil (Invitrogen) as previously described. Briefly, CHB‐PNALT‐Exo was mixed with 1 μmol/L Dil, and the exosome‐dye suspension was incubated for 5 minutes with regular mixing. After incubation, excess dye was removed from the labelled by ultracentrifugation at 100 000 *g* for 1 hours at 4°C in a 70 Ti rotor (Beckman Coulter), and the exosome pellets were washed three times by resuspension in PBS. The final pellets were resuspended in PBS. The Dil‐labelled exosomes were co‐cultured with HepG2.2.15 cells for 6 hours. Then, the HepG2.2.15 cells were washed with PBS and fixed with 4% paraformaldehyde (PFA), and uptake was observed by fluorescence microscopy.

### 2.5 Vectors and cell transfection

The pcDNA3.1 empty vector (vector) and transcription factor 21 (TCF21) and hedgehog‐interacting protein (HHIP) pcDNA3.1 expression vectors were designed and constructed by Sangon Biotech Co., Ltd., and TCF21 and HHIP small interfering RNAs (siTCF21 and siHHIP, respectively) and negative control siRNA (siNC) were purchased from Thermo Fisher Scientific. miR‐25‐3p mimics, miR‐25‐3p inhibitors, mimics NC and inhibitor NC were obtained from Sigma‐Aldrich (Merck KGaA). Cell was transfected using Lipofectamine^®^ 2000 reagent (Thermo Fisher Scientific, Inc) at 37°C with 10 nmol/L of vectors, 40 nmol/L of siRNA and/or 40 nmol/L of miRNA.

### 2.6 Cell apoptosis and viability assays

Cells were stained with annexin V and propidium iodide reagents (Annexin V‐FITC/PI Apoptosis Detection Kit) to assess apoptosis. Data were analysed using a FACSCalibur flow cytometer and BD CellQuest Pro software 5.1 (BD Biosciences).

Cells (2 × 10^4^ per well) had been planted into the 96‐well plate. Cell viability was examined according to the CCK‐8 assay following the manufacturer's protocol (Beyotime).

### 2.7 Invasion and migration assays

Cell suspension (100 µL, 5 × 10^5^/mL contained in FBS‐free RPMI‐1640) had been added into upper transwell chamber (with the pore size of 8 µm), while medium (600 µL) supplemented with 10% FBS had been added into lower transwell chamber. Image‐Pro Plus version 6 (Media Cybernetics, Inc) was used for cell counting.

Migratory capacity of HepG2.2.1.5 cells under various treatments was evaluated through scratch assay. Cells (5 × 10^5^/mL) had been cultivated within the 12‐well plates for 24 hours. Afterwards, a wound was created by scratching the plate with the pipette tip (200 µL). The wound size was determined, and photographs were taken with the microscope to compare the cell motility. The CKX41 inverted light microscope (Olympus Corporation) was used for image capture.

### 2.8 Colony formation assay

Cells under various treatments had been subjected to trypsin digestion to prepare the single‐cell suspension, which was then planted to the 6 mm incubation plates at 250 cells/well. Thereafter, cells had been cultivated for 14 days before 25 minutes of fixation with glacial acetic acid and methanol (at 1:7) at 25°C and 0.1% crystal violet staining. Colonies containing over 50 cells had been calculated by Image‐Pro Plus 6.0 (Media Cybernetics, Inc).

### 2.9 Xenograft tumour model

HepG2.2.15 cells treated with CHB‐PNALT‐Exo containing inhibitors were trypsinized, washed and resuspended in DMEM without FBS. Then, 16 male athymic nude mice (SLAC Laboratory Animal Center, Shanghai, China) were randomly divided into four groups (4 mice/group), and 2 × 10^6^ cells were subcutaneously injected into the right armpit of each mice. After the tumour formed (at 1‐2 weeks), tumour size was evaluated every 3‐4 days. At 21 days after injection, the mice were euthanized, and the excised tumour tissues were formalin‐fixed and paraffin‐embedded. All animal experiments were approved by the Animal Care and Use Committee of Central South University.

### 2.10 Tissue immunohistochemistry

Paraffin‐embedded were fixed with 4% paraformaldehyde overnight at room temperature and embedded in a paraffin block. Paraffin‐embedded slides were deparaffinized and rehydrated in a series of ethanol solutions. After two washes with PBS for 5 minutes each, antigen retrieval was performed in Citrate Antigen Retrieval Solution (Beyotime) by boiling for 10 minutes. After cooling down, slides were blocked with 10% foetal bovine serum in PBS for 1 hours. Then, various primary antibodies (Ki67, C CASP3 and E‐cadherin) were applied in a concentration of 8 μg/mL overnight at 4°C. After washed with PBS, HRP‐conjugated secondary antibodies were added on the slides for incubating 1 hour. DAB substrate solution was used to reveal the colour of antibody staining. The intensity was scored as follows: 0, none; 1, weak; 2, moderate; and 3, intense.

### 2.11 Co‐immunoprecipitation (Co‐IP)

Co‐immunoprecipitation was performed as described previously. Both the input and IP samples were analysed by Western blotting using various antibodies at the following dilutions: TCF21 antibody (1:1000), HHIP antibody (1:1000), Flag‐tag antibody (1:1000), HA‐tag antibody (1:1000) and normal rabbit/mouse IgG (CST).

### 2.12 Microarray data and identification of differentially expressed genes

The GSE101728 data set, which includes seven tumours and adjacent tissues, was downloaded from the GEO database. The differentially expressed genes (DEGs) obtained from the data set were screened using the GEO2R online tool, and RNA‐Seq data from 424 liver cancers were downloaded from the Cancer Genome Atlas (TCGA) database. The DEGs obtained from TCGA data set were screened using the “Deseq2” package in R. A log fold change (logFC)> 1 and adj. *P*‐value < .05 were considered statistically significant.

### 2.13 Predication of the target genes of miR‐25‐3p

TargetScan (http://www.targetscan.org) and EVmiRNA (http://bioinfo.life.hust.edu.cn/EVmiRNA/#!/) databases were used to predict the target genes of miR‐25‐3p.

### 2.14 Construction of PPI network

COEXPEDIA database was used to construct the PPI network.

### 2.15 Functional annotation enrichment

GO provides three categories of defined terms, including biological process (BP), cellular component (CC) and molecular function (MF) categories. GO term analysis was performed using the Database for Annotation, Visualization and Integrated Discovery (DAVID; http://david.ncifcrf.gov) online tool. *P* < .05 was set as the cut‐off criterion.

### 2.16 Human protein atlas

The direct comparison of protein expression of TCF21 between human normal and liver cancer tissues was performed by immunohistochemistry image, and direct comparison of protein expression of HHIP between human normal liver tissues and other cancers was performed by immunohistochemistry image and immunofluorescence image based on the Human Protein Atlas (https://www.proteinatlas.org).

### 2.17 UALCAN and UCSC databases

UALCAN (http://ualcan.path.uab.edu) and UCSC (http://xena.ucsc.edu/) databases were used to analyse the mRNA expression of overlapping genes in primary liver cancer tissues and their association with clinicopathological parameters.

### 2.18 Luciferase reporter assay

miR‐25‐3p mimics/inhibitors and the luciferase reporter vector containing wild‐type (WT) or mutant (MUT) 3′‐UTR of TCF21/HHIP were co‐transfected into 293T cells. After cell transfection for 48 hours, the cells were lysed and the luciferase activity was detected using the Dual‐Luciferase Assay Kit (Promega Corporation).

### 2.19 Quantitative RT‐PCR (qRT‐PCR)

The TRIzol reagent was used to extract total RNA in accordance with manufacturer protocols. RNA level was calculated by the spectrophotometer (Beckman Instruments). The PrimeScript RT Reagent Kit was utilized to reversely transcribe 2 μg RNA. Later, the LightCycler Real‐Time PCR System (Roche 480) was utilized for qRT‐PCR. All results were presented in the form of fold difference compared with actin level according to the 2^−ΔΔCt^ method. The primers are shown in Table 3. GAPDH and U6 were utilized to be the internal reference.

**TABLE 3:** The primers of miR‐25‐3p, TCF21 and HHIP

Gene	Primers
miR‐25‐3p	Forward: 5′‐CAUUGCACUUGUCUCGGUCU‐′3 Reverse: 5′‐CUCAACUGGUGUCGUGGA‐′3
TCF21	Forward: 5′‐ACCCTCTTCCTCGCTTTCTC‐′3 Reverse: 5′‐ GTGCTCTCGTTGGAAGTCAC‐′3
HHIP	Forward: 5′‐AGAAGGTGCCTGAATGGGAA‐′3 Reverse: 5′‐CTTATTCTCCAGGCGCCCTA‐′3
U6	Forward: 5′‐CTCGCTTCGGCAGCACA‐′3 Reverse: 5′‐AACGCTTCACGAATTTGCGT‐′3
GAPDH	Forward: 5′‐GTTCGTCATGGGTGTGAACC‐′3 Reverse: 5′‐CTAAGCAGTTGGTGGTGCAG‐′3

### 2.20 Western blotting

The whole‐cell protein extracts from cells or tissues were prepared using RIPA lysis buffer (Beyotime, Shanghai, China). Afterwards, the BCA Protein Assay Kit (Beyotime) was used to measure the concentration of the collected total protein. Equivalent volume of lysate protein was loaded for SDS‐PAGE isolation, which was then transferred to the PVDF membranes. Then, the membranes were incubated within the blocking solution containing 5% non‐fat milk (Sigma), followed by immunoblotting using anti‐Ki67 (1:800), anti‐TCF21 (1:500), anti‐cleaved caspase‐3 (1:800), anti‐cleaved caspase‐9 (1:1000), anti‐HHIP (1:1000), anti‐E‐cadherin (1:600) and anti‐GAPDH (1:1000) at 4°C overnight. Later, the PVDF membranes were rinsed before 2 hours of incubation with specific secondary antibodies (diluted at 1:1000) under ambient temperature, and then, membranes would be rinsed with TBST thrice. Then, the Enhanced Chemiluminescence (ECL) Western Blotting Detection Kit (Amersham Pharmacia Biotech) was utilized to detect the immunobands. All films were canned by the Bio‐Rad Molecular Imager in combination with the Image Lab Software. The ImageJ analyser software was adopted to analyse the relative protein band densities. Each protein band density was standardized based on GAPDH.

### 2.21 Statistical analyses

For miR‐25‐3p, a relative difference in miR‐25‐3p expression > 2.7 was considered to be high expression. The correlations of miR‐25‐3p expression with various clinicopathological features were examined using the chi‐square test. Survival curves were constructed using the Kaplan‐Meier method and analysed by the log‐rank test. Survival among patients with different miR‐25‐3p mRNA expression levels in liver tumours was compared using the univariate Cox proportional hazards model. All values have been presented as the means ± standard deviation (SD). All statistical analyses were performed using SPSS software (SPSS 19.0, IBM Inc). ANOVA followed by LSD test was performed for multiple comparisons, and the Student's *t* test was used for comparisons between two groups. A difference with a *P* < .05 was considered statistically significant.

## 3 RESULTS

### 3.1 CHB‐PNALT‐Exo (≥A2) promotes cell proliferation, invasion and migration, and inhibits apoptosis in HepG2.2.15 cells, and miR‐25‐3p is closely related to the poor survival in HBV‐positive patients with liver cancer

**FIGURE 1:**
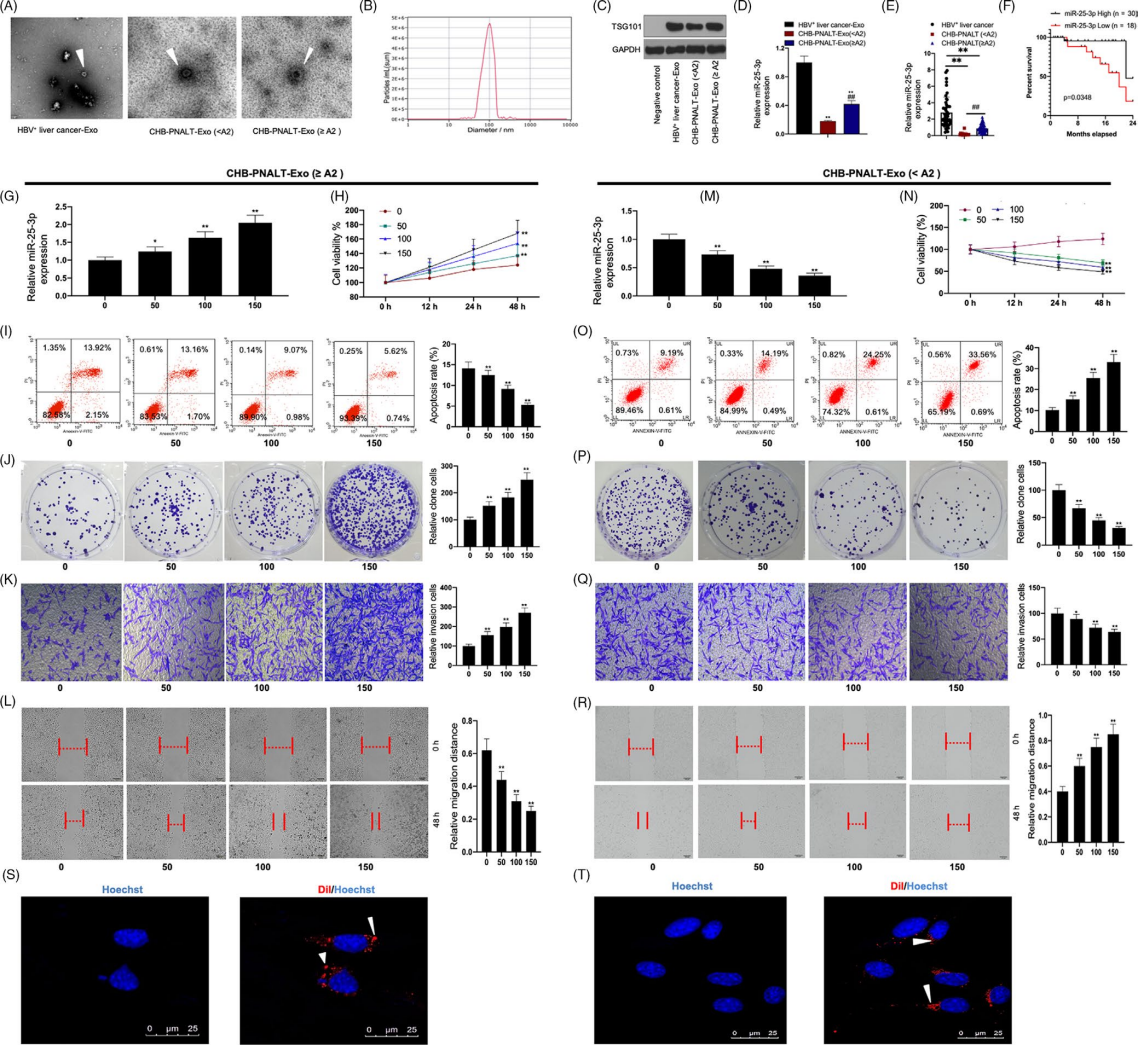
CHB‐PNALT‐Exo regulated the proliferation, invasion and migration, and inhibited the apoptosis in HepG2.2.15 cells. A, The morphology of exosomes obtained from HBV‐positive patients with liver cancer, CHB‐PNALT (<A2) and CHB‐PNALT (≥A2) was observed under scanning electron microscope. B, Exosomes' diameter was detected. C, Western blot assay was used to determine TSG101 protein expression. D, E, RT‐PCR assay was used to detect miR‐25‐3p expression. F, Kaplan‐Meier analysis was used to show survival rate of HBV‐positive patients with liver cancer between overexpression of miR‐25‐3p and inhibition of miR‐25‐3p. After HepG2.2.15 cells were incubated with 50, 100 and 150 μg/mL of CHB‐PNALT‐Exo (≥A2), miR‐25‐3p expression, cell viability, apoptosis, invasion and migration were detected by RT‐PCR assay (G), CCK‐8 assay (H), flow cytometry analysis (I), clone formation assay (J), transwell assay (K) and scratch‐wound assay (L). After HepG2.2.15 cells were incubated with 50, 100 and 150 μg/mL of CHB‐PNALT‐Exo (<A2), miR‐25‐3p expression, cell viability, apoptosis, invasion and migration were detected by RT‐PCR assay (M), CCK‐8 assay (N), flow cytometry analysis (O), clone formation assay (P), transwell assay (Q) and scratch‐wound assay (R). (S, T) Uptake of CHB‐PNALT‐Exo (≥A2) or CHB‐PNALT‐Exo (<A2) was observed in HepG2.2.15 cells (Dil‐Exo; Dil is shown in red); nuclei were stained with Hoechst 33 342 (blue). Arrowheads indicate Dil‐labelled exosomes within HepG2.2.15 cells, scale bars: 50 μm. GAPDH or U6 was used as a load control. Data are presented as the mean ± standard deviation. **P < .01 vs Con group

Exosomes were isolated from the peripheral blood of HBV‐positive patients with liver cancer and CHB patients with PNALT and liver inflammation grade ≥ A2 or <A2 (Figure 1A). The volume kurtosis of the exosomes' diameter was 10 nm‐100 nm (Figure 1B). Expression of the exosome biomarker TSG101 was assessed by Western blotting assay (Figure 1C). The analysis showed that miR‐25‐3p expression in CHB‐PNALT‐Exo (≥A2) was lower than that in HBV‐positive liver‐Exo and was higher than that in CHB‐PNALT‐Exo (<A2) group (Figure 1D). Likewise, miR‐25‐3p expression in the CHB‐PNALT (≥A2) group was lower than that in the HBV‐positive liver group and was higher than that in the CHB‐PNALT (<A2) group (Figure 1E). We also found that miR‐25‐3p expression was significantly correlated with tumour size, tumour number, tumour differentiation, vascular invasion and TNM stage (*P *< .05; Table 2), but was not significantly correlated with gender, age or AFP level (Table 2). The Kaplan‐Meier analysis revealed that patients with tumours that overexpressed miR‐25‐3p had shorter tumour‐free survival than HBV‐positive patients with liver cancer (Figure 1F). Univariate Cox regression analysis showed that the miR‐25‐3p expression level and vascular invasion were closely related to overall survival (Table 4). As the mount of CHB‐PNALT‐Exo (≥A2) increased in the HepG2.2.15 cells, miR‐25‐3p expression and cell viability increased, and cell apoptosis was decreased (Figure 1G‐I). Our results further showed that the proliferation, invasion and migration were significantly increased in HepG2.2.15 cells incubated with CHB‐PNALT‐Exo (≥A2) (Figure 1J‐L). In contrast, as the amount of CHB‐PNALT‐Exo (<A2) in HepG2.2.15 cells increased, miR‐25‐3p expression and cell viability were decreased, and cell apoptosis was increased (Figure 1M‐O). Our results further showed that the proliferation, invasion and migration of HepG2.2.15 cells incubated with CHB‐PNALT‐Exo (<A2) were significantly decreased (Figure 1P‐R). Interestingly, 100 μg/mL of CHB‐PNALT‐Exo markedly promoted cell proliferation and metastasis. Exosome uptake clearly observed HepG2.2.15 cells, which were incubated with Dil‐labelled exosomes (100 μg/mL) from CHB‐PNALT (≥A2) and (<A2) patients (Figure 1S,T). Therefore, 100 μg/mL of CHB‐PNALT‐Exo was used to perform the following experiments. Therefore, 100 μg/mL of CHB‐PNALT‐Exo were used at 100 μg/mL in subsequent experiments.

**TABLE 4:** Univariate Cox proportional hazards analyses of miR‐25‐3p expression and overall survival for patients with liver cancer

Variable	*P* value	HR	95% CI
Gender (Female vs male)	.1284	1.4301	0.9017‐2.268
Age (≥50 vs <50)	.5405	0.8551	0.5181‐1.412
AFP (ng/mL) (>400 vs ≤400)	.8094	1.0616	0.6531‐1.726
miR‐25‐3p expression (high vs low)	.0042**	3.1205	1.4314‐6.803
Tumour size (cm) (>5 vs ≤5)	.7671	1.0785	0.6538‐1.779
Tumour number (Single vs multiple)	.8004	0.9401	0.5823‐1.518
Vascular invasion (Yes vs No)	.0254**	1.9133	1.0834‐3.379
Tumour differentiation (I‐II vs III‐IV)	.1311	0.6605	0.3855‐1.132
TNM stage			
I vs II	.9911	1.0032	0.5663‐1.777
II vs III	.2998	1.4049	0.7388‐2.671

Abbreviations: CI, confidence interval; HR, hazard ratio; TNM, tumour‐node‐metastasis.

** *P* < .01.

### 3.2 miR‐25‐3p regulates the viability, apoptosis, proliferation, invasion and migration of HepG2.2.15 cells

**FIGURE 2:**
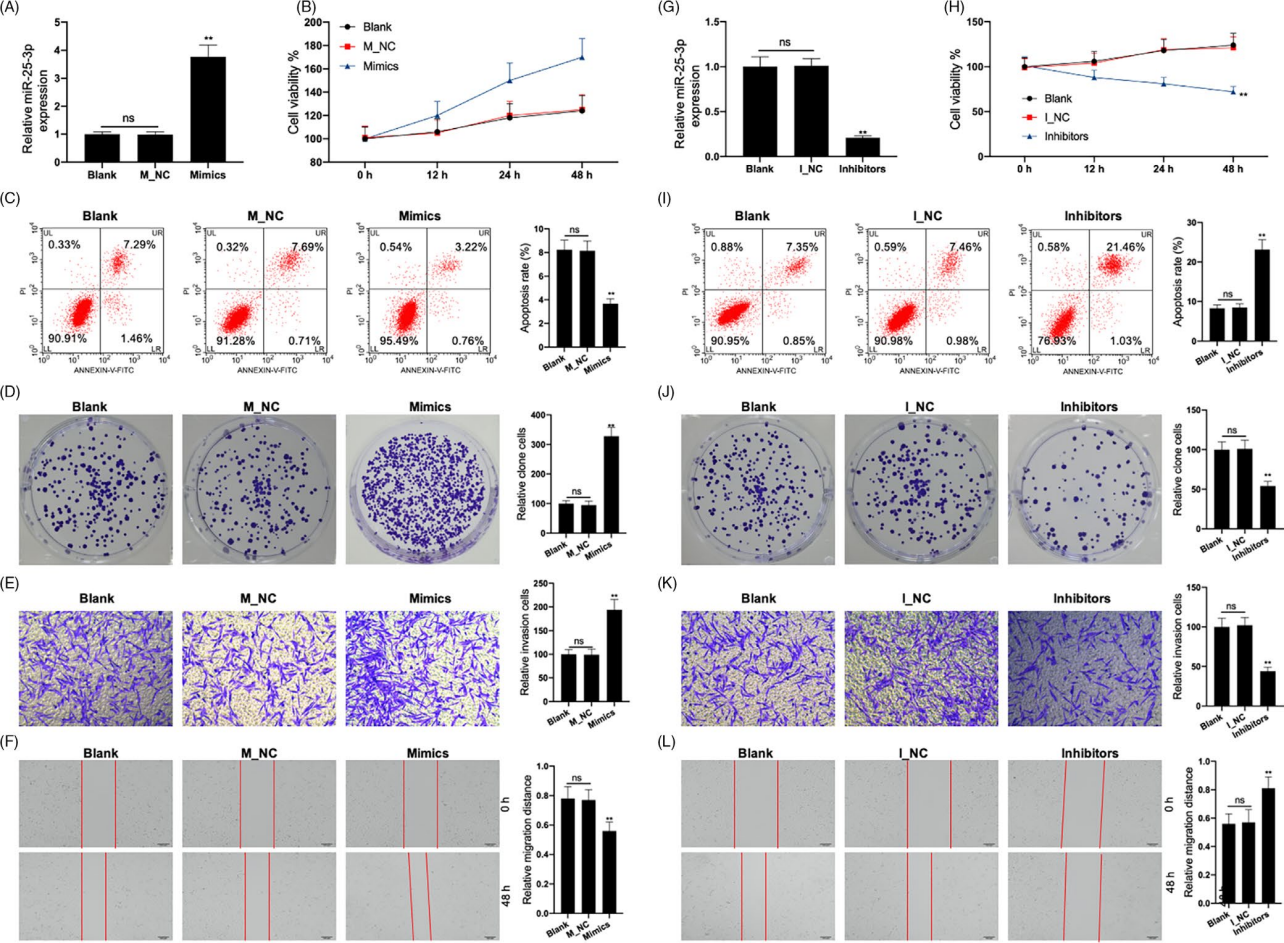
miR‐25‐3p regulated the viability, apoptosis, proliferation, invasion and migration of HepG2.2.15 cells. After miR‐25‐3p mimics were transfected into HepG2.2.15 cells, miR‐25‐3p expression, cell viability, apoptosis, invasion and migration were detected by RT‐PCR assay (A), CCK‐8 assay (B), flow cytometry analysis (C), clone formation assay (D), transwell assay (E) and scratch‐wound assay (F). After miR‐25‐3p inhibitors were transfected into HepG2.2.15 cells, miR‐25‐3p expression, cell viability, apoptosis, invasion and migration were detected by RT‐PCR assay (G), CCK‐8 assay (H), flow cytometry analysis (I), clone formation assay (J), transwell assay (K) and scratch‐wound assay (L). U6 was used as a load control. Data are presented as the mean ± standard deviation. **P < .01 vs Blank group

In HepG2.2.15 cells transfected with the miR‐25‐3p mimic, miR‐25‐3p expression was upregulated (Figure 2A). This overexpression of miR‐25‐3p increased cell viability and decreased apoptosis, as assessed by CCK‐8 and flow cytometry assays (Figure 2B,C). The results of clone formation, transwell and scratch‐wound assays showed that overexpression of miR‐25‐3p promoted cell proliferation, invasion and migration (Figure 2D‐F). In HepG2.2.15 cells transfected with the miR‐25‐3p inhibitor, miR‐25‐3p expression was knocked down (Figure 2G). Knockdown of miR‐25‐3p suppressed cell viability and induced apoptosis as shown by the CCK‐8 and flow cytometry assays (Figure 2H,I). The results of clone formation, transwell and scratch‐wound assays showed that knockdown of miR‐25‐3p inhibited cell proliferation, invasion and migration (Figure 2J‐L).

### 3.3 Abolishment of the proliferation‐, invasion‐ and migration‐promoting and apoptosis‐inhibiting functions of CHB‐PNALT‐Exo (≥A2) in HepG2.2.15 cells by knockdown of miR‐25‐3p

**FIGURE 3:**
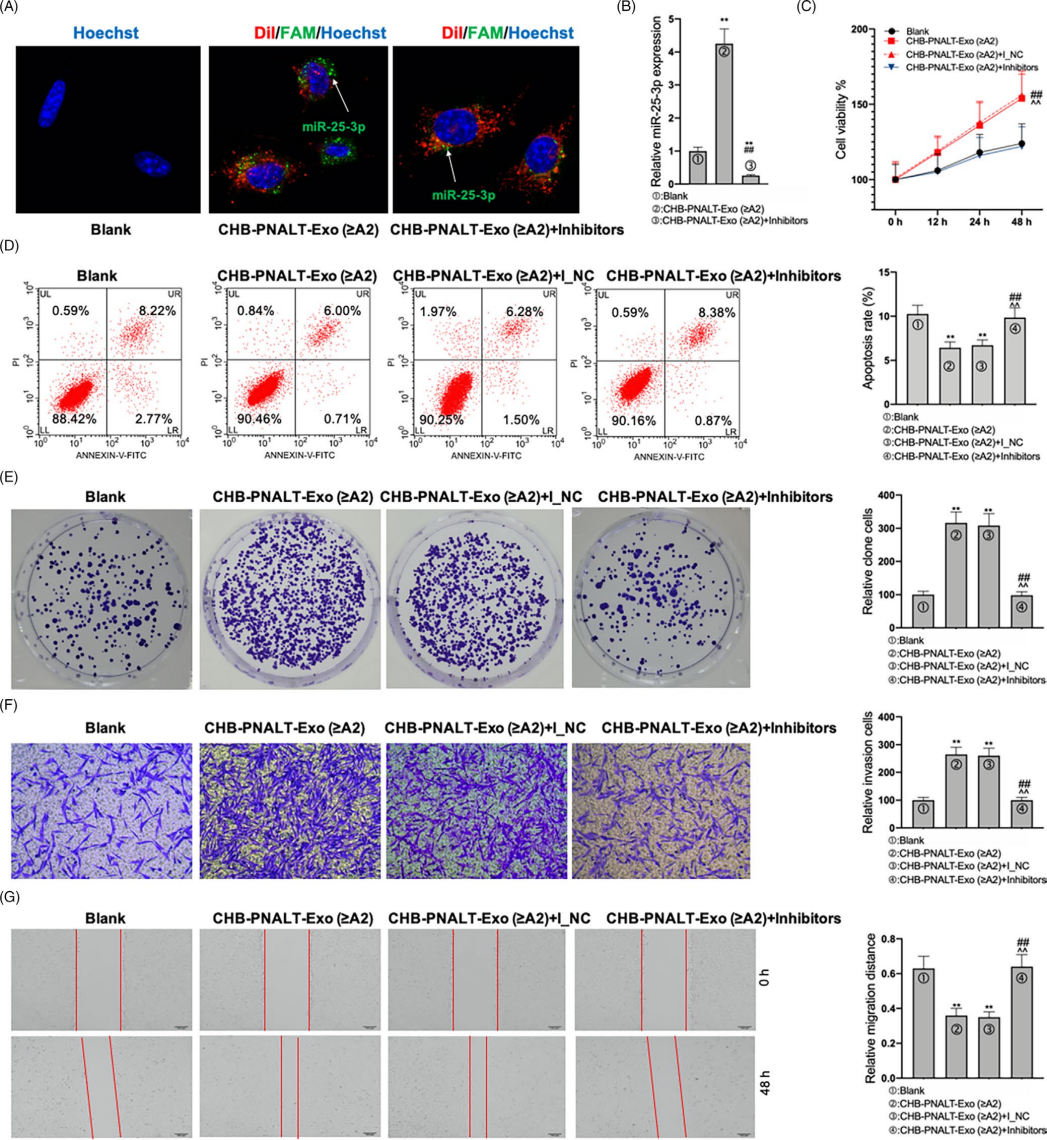
The functions of CHB‐PNALT‐Exo (≥A2) promoting the proliferation, invasion and migration, and inhibiting the apoptosis in HepG2.2.15 cells were abolished by inhibition of miR‐25‐3p. A, Exosomes derived from CHB‐PNALT‐Exo (≥A2) transfected with FAM‐miR‐25‐3p were labelled with Dil and then added to HepG2.2.15. FAM‐miR‐25‐3p signals are green, and Dil is red. Nuclei were stained with Hoechst 33 342 (blue). Arrowheads indicate FAM‐miR‐25‐3p in cytoplasm of HepG2.2.15 cells. Scale bars: 50 μm. B, RT‐PCR assay was used to detect miR‐25‐3p expression. After HepG2.2.15 cells were treated with CHB‐PNALT‐Exo (≥A2) or CHB‐PNALT‐Exo (≥A2) containing miR‐25‐3p inhibitor NC or miR‐25‐3p inhibitors, cell viability, apoptosis, invasion and migration were detected by CCK‐8 assay (C), flow cytometry analysis (D), clone formation assay (E), transwell assay (F) and scratch‐wound assay (G). U6 was used as a load control. Data are presented as the mean ± standard deviation. **P < .01 vs Blank group, ##*P* < .01 vs CHB‐PNALT‐Exo (≥A2) group and ^^*P* < .01 vs CHB‐PNALT‐Exo (≥A2) + I_NC group

Exosomes derived from CHB‐PNALT (≥A2) patients transfected with FAM‐labelled miR‐25‐3p were labelled with Dil and then mixed with HepG2.2.15 cells, and exosomes derived from CHB‐PNALT (≥A2) containing miR‐25‐3p inhibitors transfected with FAM‐labelled miR‐25‐3p were labelled with Dil and then mixed with HepG2.2.15 cells (Figure 3A). qRT‐PCR assay showed that miR‐25‐3p levels were increased in CHB‐PNALT‐Exo (≥A2) and decreased in CHB‐PNALT‐Exo (≥A2) containing miR‐25‐3p inhibitors (Figure 3B). The assay results showed that the cell viability, proliferation, invasion and migration of the CHB‐PNALT‐Exo (≥A2) cells were higher than those in the blank group, whereas cell apoptosis was lower in the CHB‐PNALT‐Exo (≥A2) cells than that in the blank group (Figure 3C‐G). The results of CCK‐8, flow cytometry, clone formation, transwell and scratch‐wound assays showed that knockdown of miR‐25‐3p blocked the cell viability‐, proliferation‐, invasion‐ and migration‐promoting and cell apoptosis‐inhibiting effects of CHB‐PNALT‐Exo (≥A2).

### 3.4 Reversal of the in vivo tumour growth‐promoting functions of CHB‐PNALT‐Exo (≥A2) by knockdown of miR‐25‐3p

**FIGURE 4:**
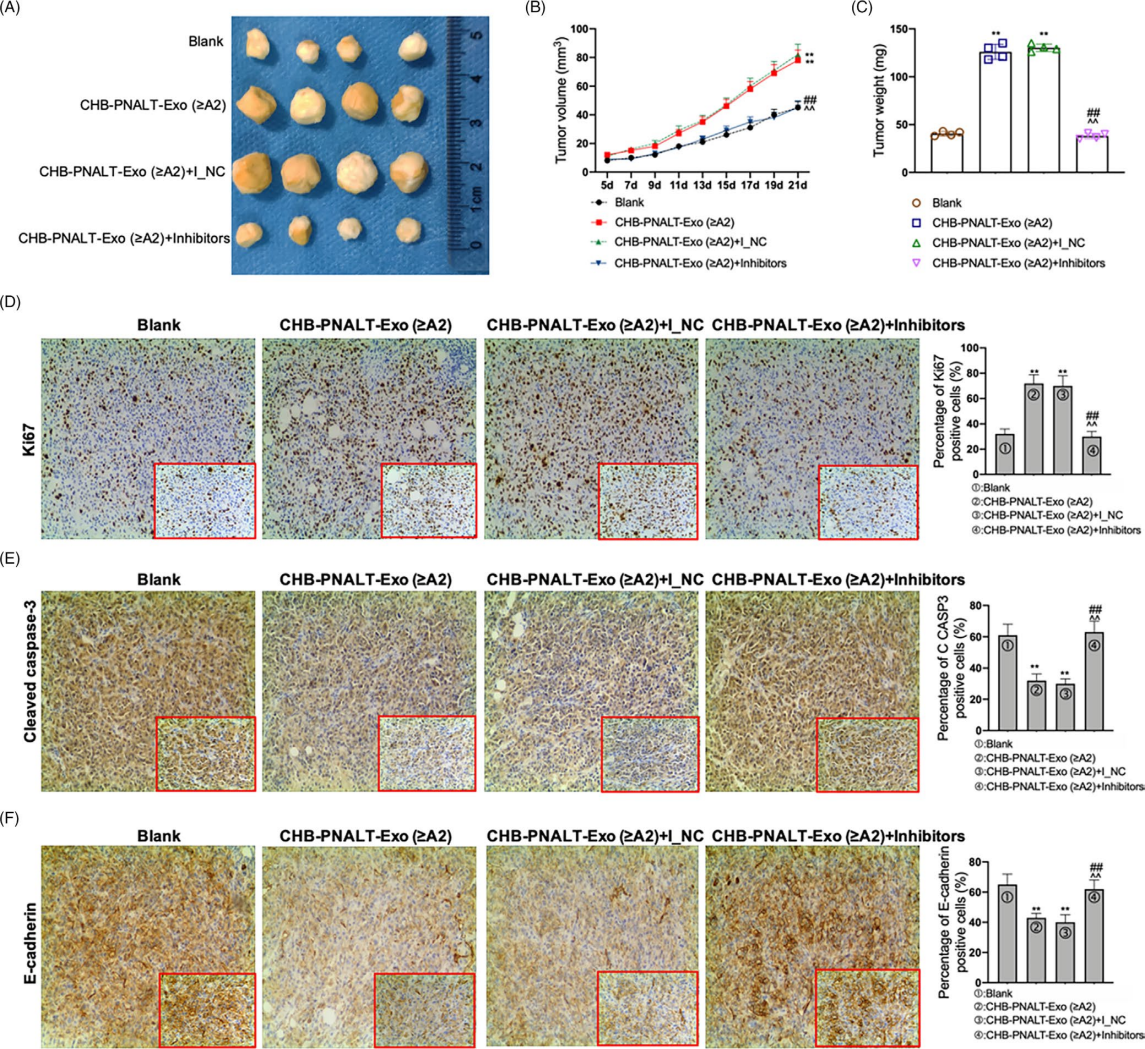
The functions of CHB‐PNALT‐Exo (≥A2) promoting tumour growth in *vivo* were reversed by inhibition of miR‐25‐3p. HepG2.2.15 cells with CHB‐PNALT‐Exo (≥A2) or CHB‐PNALT‐Exo (≥A2) containing miR‐25‐3p inhibitor NC/miR‐25‐3p inhibitors were injected with nude mouse, respectively, (A) the photograph of tumour; (B) statistical graph of tumour volume; (C) statistical graph of tumour weight; (D) the expressions of Ki67, (E) cleaved caspase‐3 and (F) E‐cadherin was detected by IHC assay. Data are presented as the mean ± standard deviation. **P < .01 vs Blank group, ##*P* < .01 vs CHB‐PNALT‐Exo (≥A2) group and ^^*P* < .01 vs CHB‐PNALT‐Exo (≥A2) + I_NC group

A tumorigenicity assay in nude mice was performed to evaluate the effect of CHB‐PNALT‐Exo (≥A2) and CHB‐PNALT‐Exo (≥A2) containing miR‐25‐3p inhibitors on tumour growth. HepG2.2.15 cells incubated with CHB‐PNALT‐Exo (≥A2) or CHB‐PNALT‐Exo (≥A2) + inhibitor NC were injected into the nude mice. Analysis of the mice showed that after 21 days (Figure 4A), tumour weight and tumour volume were significantly increased (Figure 4B,C). In contrast, tumour weight and tumour volume were the same in the blank and CHB‐PNALT‐Exo (≥A2) + inhibitor groups. This result indicated that the miR‐25‐3p inhibitor inhibited the tumour growth‐promoting functions of CHB‐PNALT‐Exo (≥A2). Further analysis showed that Ki67 expression was upregulated and cleaved caspase‐3 and E‐cadherin expression levels were downregulated in the CHB‐PNALT‐Exo (≥A2) group when compared to the corresponding levels in the blank group. Knockdown of miR‐25‐3p decreased Ki67 expression and increased the levels of cleaved caspase‐3 and E‐cadherin expression in CHB‐PNALT‐Exo (≥A2)‐treated tumour tissues of nude mice (Figure 4D‐F).

### 3.5 Bioinformatics identification of the target genes of miR‐25‐3p

**FIGURE 5:**
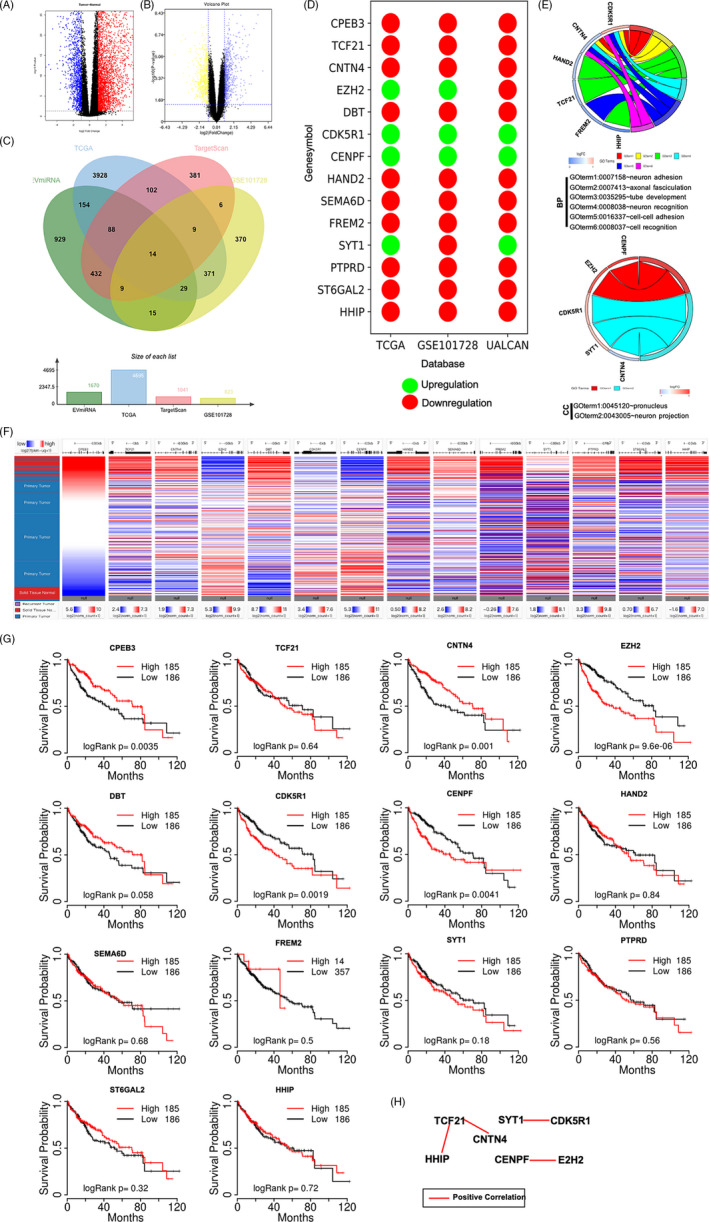
Bioinformatics identifies target genes of miR‐25‐3p. A, Volcano map of TCGA database including 424 liver cancer cases. B, Volcano map of GSE101728 data set including 7 adjacent tissues and 7 tumour tissues. C, Venn map of 14 overlapping genes from EVmiRNA, TCGA, GSE101728 and TargetScan databases. D, The expression of 14 overlapping genes was shown, red and green circles indicate downregulation and upregulation, respectively. E, GO enrichment analysis of 14 overlapping genes based on DAVID online website. F, The mRNA expression of 14 overlapping genes between human normal and liver cancer tissues was investigated based on UCSC database. G, Kaplan‐Meier analysis was used to show survival rate of patients with liver cancer between overexpression of gene and inhibition of gene. H, COEXPEDIA database was used to construct PPI network about 14 overlapping genes

A total of 4695 DEGs, including 1262 downregulated and 3433 upregulated genes, were obtained from TCGA database (Figure 5A), and 824 DEGs, including 459 downregulated and 365 upregulated genes, were obtained from the GSE101728 data set (Figure 5B). The 14 overlapping genes present in both the DEG data set and the miR‐25‐3p target genes are shown in Figure 5C. The expression of 14 overlapping genes was assessed using UALCAN (Figure 5D). The results of GO enrichment analysis using DAVID showed that these genes were mainly enriched in two terms, “BP” and “CC” (Figure 5E). A heat map of the 14 overlapping genes in human liver cancer and normal tissues was generated using UALCAN (Figure 5F). The Kaplan‐Meier analysis revealed that *CPEB3*, *CNTN4*, *EZH2*, *CDK5R1* and *CENPF* were significantly related to poor survival (Figure 5G). The co‐expression of TCF21 and HHIP was accessed using the COEXPEDIA (Figure 5H).

### 3.6 Evaluation of protein expression levels of TCF21 and HHIP in liver cancer tissues using the Human Protein Atlas

**FIGURE 6:**
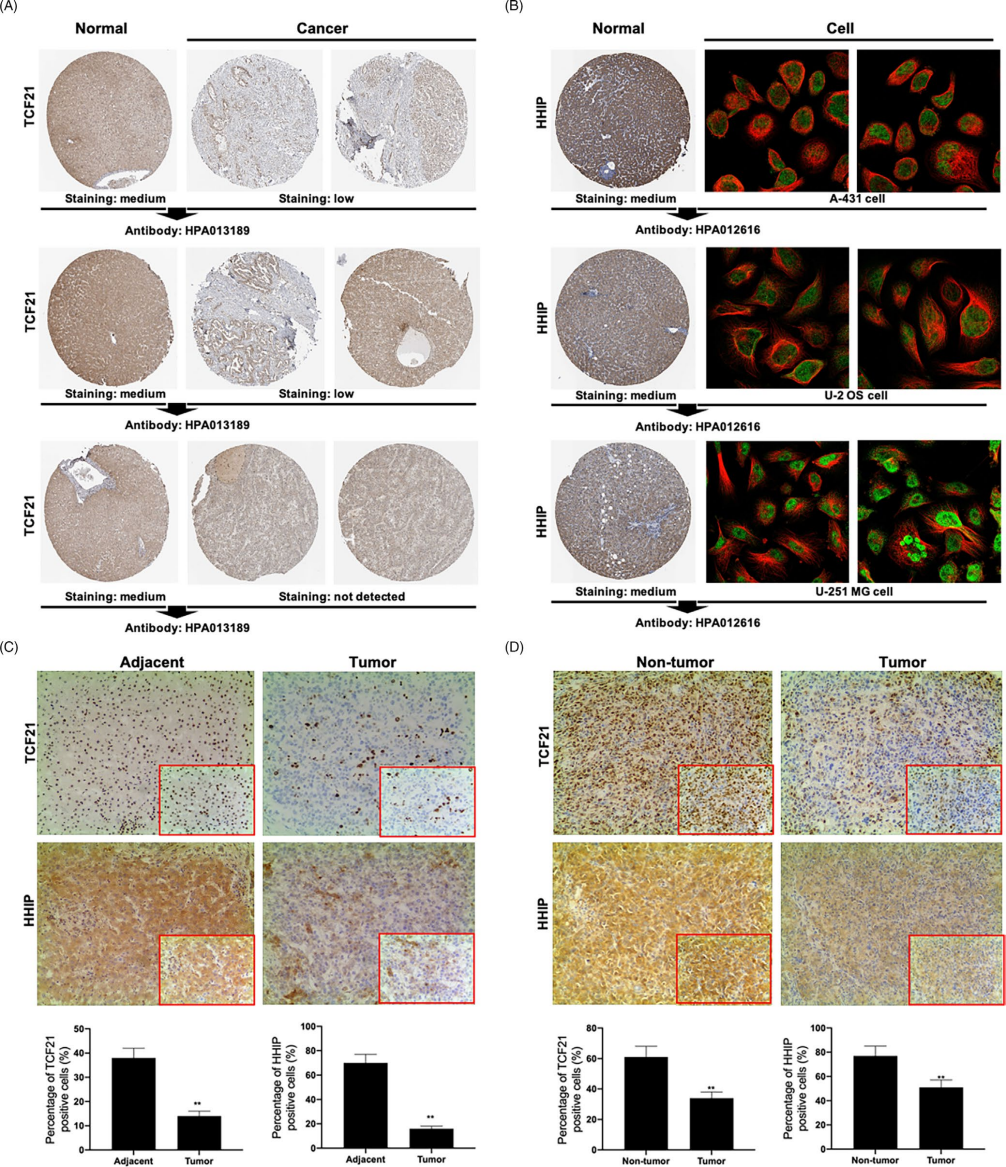
The protein expression of TCF21 and HHIP in liver cancer tissues was identified based on the Human Protein Atlas database. A, TCF21 protein expression between normal liver tissues and liver cancer tissues based on the Human Protein Atlas database. B, HHIP protein expression in normal liver tissues and mainly located on nucleoplasm of A‐431, U‐2 OS and U‐251 MG cells. C, IHC assay was used to detect TCF21 expression or HHIP expression between normal adjacent tissues and tumour tissues in patients with liver cancer. D, IHC assay was used to detect TCF21 expression or HHIP expression between non‐tumour tissues and tumour tissues in mouse

Based on analyses using the Human Protein Atlas, TCF21 protein levels were found to be low or absent in liver cancer tissues and were medium in normal liver tissues (Figure 6A). HHIP expression levels were also medium in normal liver tissues; however, HHIP expression levels in liver cancer tissues were not reported in the Human Protein Atlas database. Our analysis showed that HHIP was mainly located in the nucleoplasm of A‐431, U‐2 OS and U‐251 MG cells (Figure 6B), and TCF21 and HHIP were significantly downregulated in human tumour tissues when compared to the levels in the corresponding adjacent tissues (Figure 6C). TCF21 and HHIP expression levels were also significantly lower in tumour tissues than in the non‐tumour tissues of the mice (Figure 6D).

### 3.7 *TCF21* and *HHIP* are target genes of miR‐25‐3p

**FIGURE 7:**
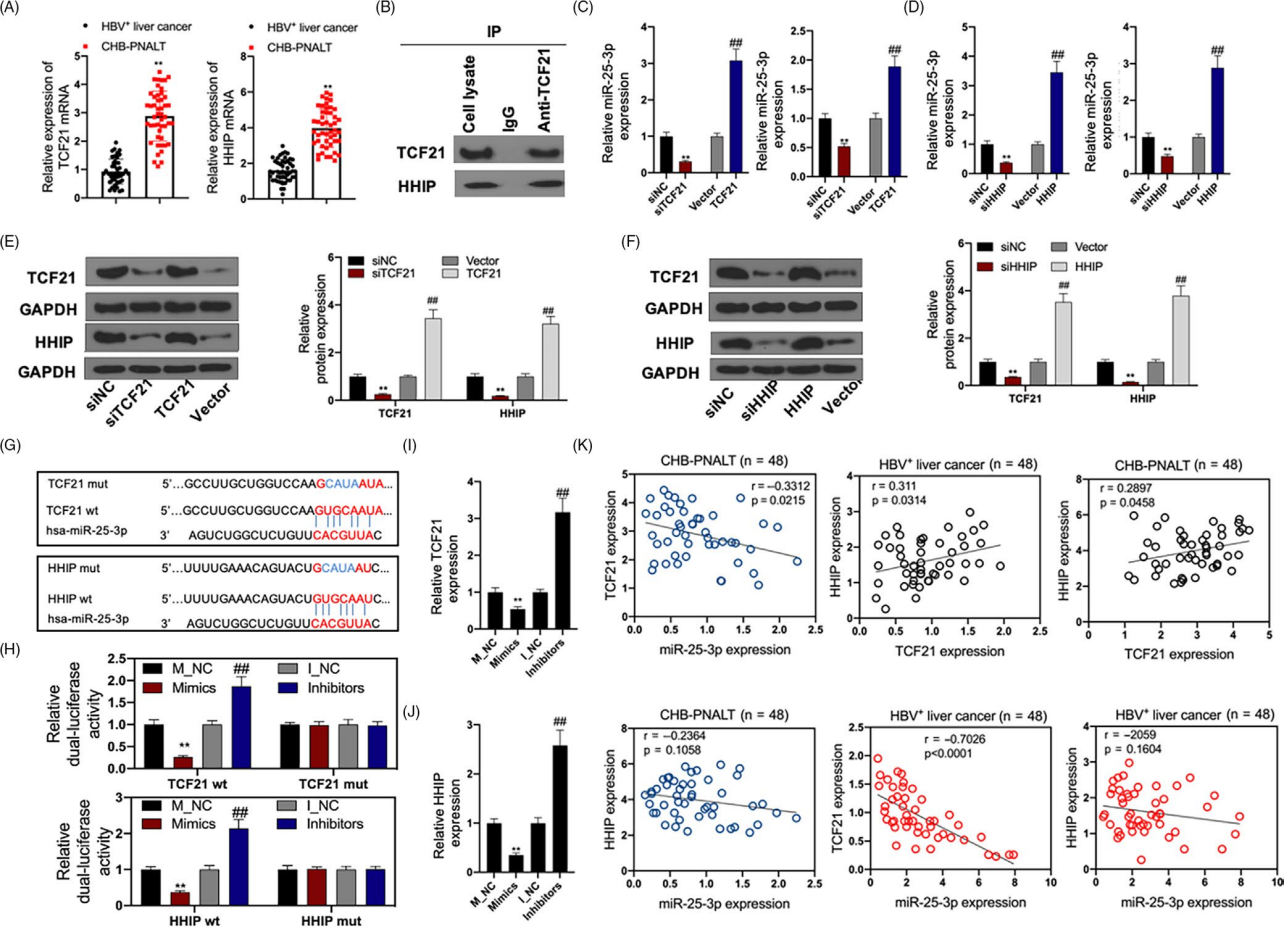
Both TCF21 and HHIP were target genes of miR‐25‐3p. A, The mRNA expression of TCF21 and HHIP in CHB patients with PNALT (≥A2) was detected by RT‐PCR assay. B, Co‐immunoprecipitation experiments indicated that TCF21 directly interact with HHIP in HepG2.2.15 cells. C, The mRNA expression of TCF21 and HHIP was detected by RT‐PCR assay in TCF21 inhibition or overexpression transfected HepG2.2.15 cells. D, The mRNA expression of TCF21 and HHIP was detected by RT‐PCR assay in HHIP inhibition or overexpression transfected HepG2.2.15 cells. E, The protein expression of TCF21 and HHIP was detected by Western blot assay in TCF21 inhibition or HHIP inhibition transfected HepG2.2.15 cells. F, The protein expression of TCF21 and HHIP was detected by Western blot assay in TCF21 overexpression or HHIP overexpression transfected HepG2.2.15 cells. G, TargetScan database showed that binding site of TCF21 or HHIP and miR‐340‐5p. H, Luciferase reporter assays were used to prove that miR‐340‐5p can target TCF21 or HHIP. I, J, The mRNA expression of TCF21 and HHIP was detected by RT‐PCR assay in miR‐25‐3p mimics or miR‐25‐3p inhibitor‐transfected HepG2.2.15 cells. K, Correlation analysis of TCF21/HHIP and miR‐25‐3p in CHB patients with PNALT (≥A2), correlation analysis of TCF21 and HHIP/miR‐25‐3p in HBV‐positive patients with liver cancer and correlation analysis of TCF21 and HHIP/miR‐25‐3p in CHB patients with PNALT (≥A2). GAPDH or U6 was used as a load control. Data are presented as the mean ± standard deviation. **P < .01 vs siNC/Vector/M‐NC group and ##*P* < .01 vs I‐NC group

The expression levels of TCF21 and HHIP were upregulated in the tissue of CHB patients with PNALT (≥A2) (Figure 7A). Co‐IP experiments indicated that TCF21 directly interacted with HHIP in HepG2.2.15 cells (Figure 7B). Inhibition of TCF21 decreased the expression of both TCF21 and HHIP, and inhibition of HHIP decreased the expression of both HHIP and TCF21. Overexpression of TCF21 increased the expression of both TCF21 and HHIP, and overexpression of HHIP increased the expression of both HHIP and TCF21 (Figure 7C‐F). The putative miR‐25‐3p binding sites and modified sequences located in the 3′ UTRs of TCF21 and HHIP are shown in Figure 7G. The luciferase activity of cells transfected with TCF21‐wt or HHIP‐wt was significantly decreased by the miR‐25‐3p mimics (Figure 7H). TCF21 and HHIP were significantly downregulated in HepG2.2.15 cells transfected with the miR‐25‐3p mimics and were upregulated in HepG2.2.15 cells transfected with the miR‐25‐3p inhibitors (Figure 7I,J). The expression of TCF21 and HHIP was negatively correlated with miR‐25‐3p expression in CHB patients with PNALT (≥A2). The expression of HHIP was significantly positively correlated with TCF21 expression in HBV‐positive patients with liver cancer and CHB patients with PNALT (≥A2). The expression of TCF21 and HHIP was negatively correlated with miR‐25‐3p expression in HBV‐positive patients with liver cancer (Figure 7K).

### 3.8 TCF21 and HHIP regulated cell viability, proliferation, apoptosis, invasion and migration in HepG2.2.15 cells

**FIGURE 8:**
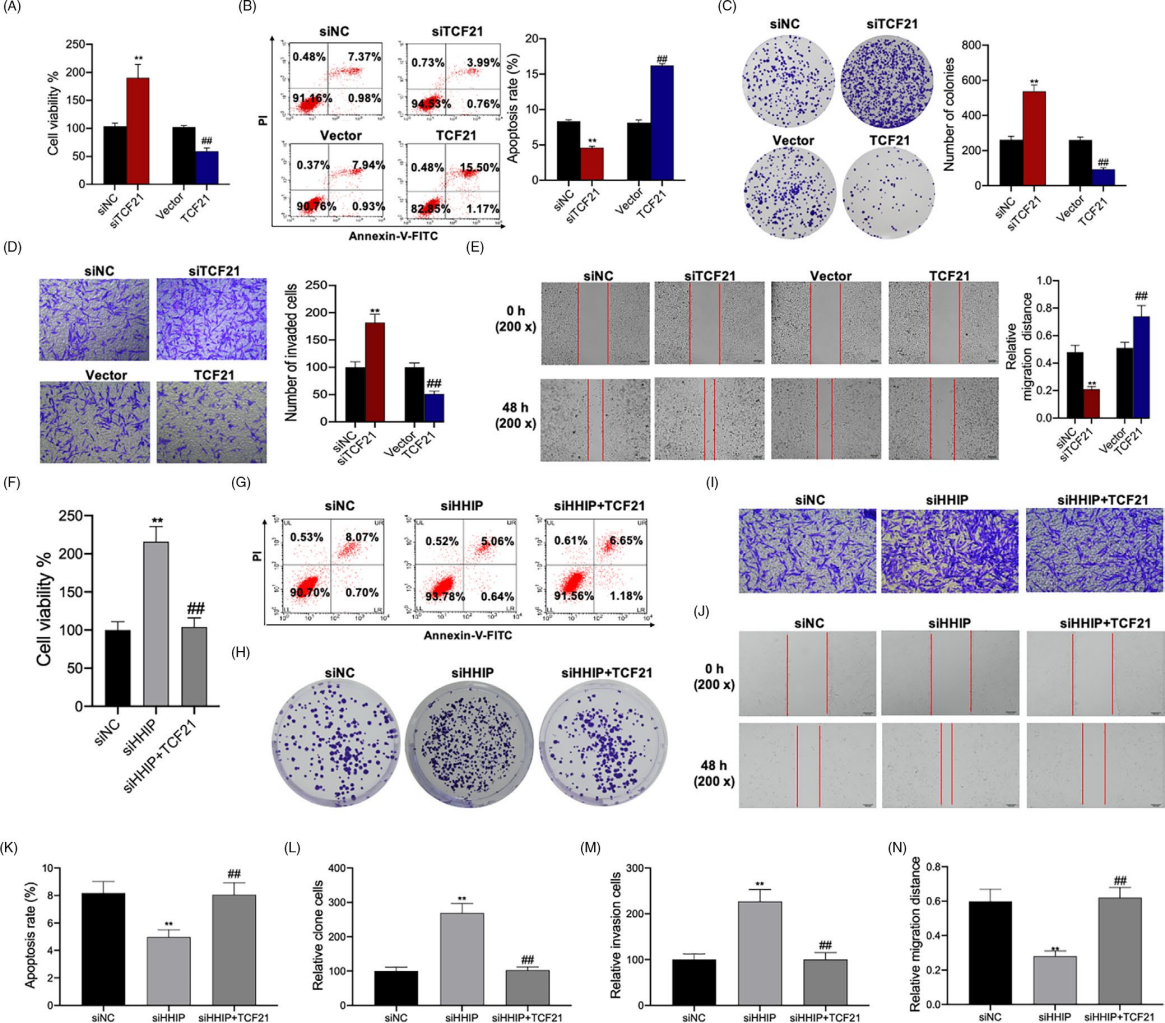
TCF21 and HHIP regulated the viability, proliferation, apoptosis, invasion and migration in HepG2.2.15 cells. After TCF21 inhibition and TCF21 overexpression transfected into HepG2.2.15 cells, (A) cell viability was detected by CCK‐8 assay, (B) cell apoptosis was detected by flow cytometry assay, (C) cell proliferation was detected by colony formation assay, (D) cell invasion was detected by transwell assay, and (E) cell migration was detected by scratch‐wound assay. After siRNA‐HHIP or siRNA‐HHIP and TCF21 overexpression transfected into HepG2.2.15 cells, (F) cell viability was detected by CCK‐8 assay, (g) cell apoptosis was detected by flow cytometry assay, (H) cell proliferation was detected by colony formation assay, (I) cell invasion was detected by transwell assay, and (J) cell migration was detected by scratch‐wound assay. K, Statistical graph of apoptosis rate. L, Statistical graph of the number of clone cells. M, Statistical graph of the number of invasion cells. N, Statistical graph of migration distance. Data are presented as the mean ± standard deviation. **P < .01 vs siNC group, ##*P* < .01 vs Vector/siHHIP group

Inhibition of TCF21 significantly increased cell viability, proliferation, invasion and migration, and decreased apoptosis in HepG2.2.15 cells. In contrast, overexpression of TCF21 significantly decreased cell viability, proliferation, invasion and migration and increased apoptosis in HepG2.2.15 cells (Figure 8A‐E). Likewise, inhibition of HHIP significantly increased cell viability, proliferation, invasion and migration and decreased apoptosis in HepG2.2.15 cells. Overexpression of TCF21 reversed the effects of TCF21 inhibition, thus decreasing cell viability, proliferation, invasion and migration, and increasing apoptosis in HepG2.2.15 cells (Figure 8F‐N).

### 3.9 The blocking effects of miR‐25‐3p inhibitors on the effect of CHB‐PNALT‐Exo (≥A2) on cell proliferation and metastasis in HepG2.2.15 cells are reversed by knockdown of TCF21 and HHIP

**FIGURE 9:**
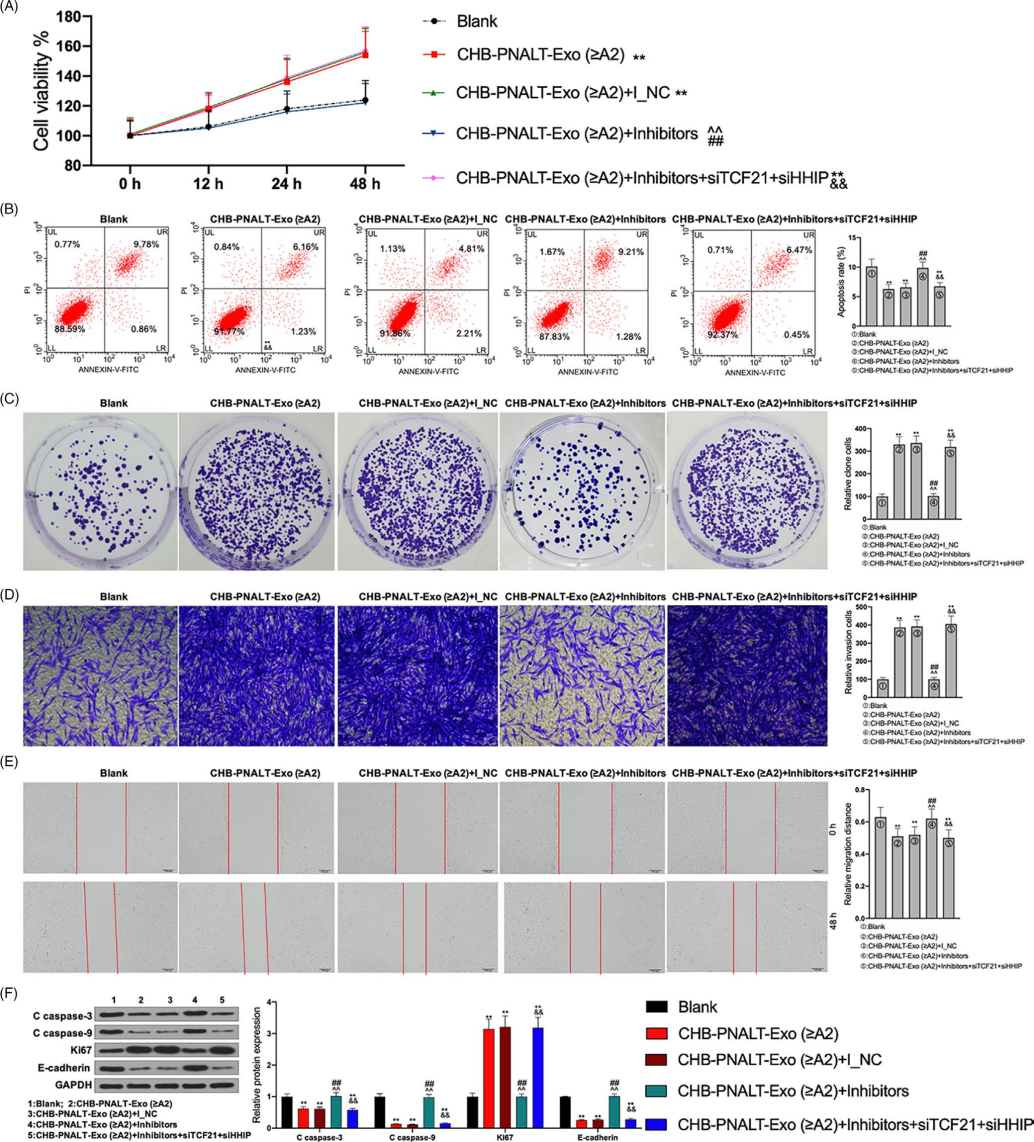
The functions of miR‐25‐3p inhibitors abolishing the effects of CHB‐PNALT‐Exo (≥A2) on the proliferation and metastasis were reversed by knockdown of TCF21 and HHIP in HepG2.2.15 cells. After siRNA‐TCF21 and siRNA‐HHIP were transfected into CHB‐PNALT‐Exo (≥A2) containing miR‐25‐3p inhibitor‐treated HepG2.2.15 cells, cell viability, apoptosis, invasion, migration and the expression of cleaved caspase‐3/‐9, Ki67 and E‐cadherin were detected by CCK‐8 assay (A), flow cytometry analysis (B), clone formation assay (C), transwell assay (D), scratch‐wound assay (E) and Western blot assay (F). GAPDH was used as a load control. Data are presented as the mean ± standard deviation. **P < .01 vs Blank group, ##*P* < .01 vs CHB‐PNALT‐Exo (≥A2) group, ^^*P* < .01 vs CHB‐PNALT‐Exo (≥A2) + I_NC group and &&*P* < .01 vs CHB‐PNALT‐Exo (≥A2) + inhibitors group

A CCK‐8 assay showed that the viability of HepG2.2.15 cells treated with CHB‐PNALT‐Exo (≥A2) containing miR‐25‐3p inhibitors was lower than that of HepG2.2.15 cells treated with CHB‐PNALT‐Exo (≥A2) (Figure 9A). Knockdown of TCF21 and HHIP abolished the viability‐, proliferation‐, invasion‐ and migration‐suppressing and apoptosis‐inducing effects of miR‐25‐3p inhibitors in CHB‐PNALT‐Exo (≥A2)‐treated HepG2.2.15 cells (Figure 9A‐E). The levels of cleaved caspase‐3/‐9 and E‐cadherin were decreased by CHB‐PNALT‐Exo (≥A2) and CHB‐PNALT‐Exo (≥A2) containing inhibitor NC, and Ki67 expression was increased by CHB‐PNALT‐Exo (≥A2) and CHB‐PNALT‐Exo (≥A2) containing inhibitor NC. The cleaved caspase‐3/‐9 and E‐cadherin expression‐suppressing and Ki67 expression‐promoting functions of CHB‐PNALT‐Exo (≥A2) and CHB‐PNALT‐Exo (≥A2) containing inhibitor NC were reversed by miR‐25‐3p inhibitors. In addition, knockdown of TCF21 or HHIP abolished the cleaved caspase‐3/‐9, Ki67 and E‐cadherin expression‐regulating functions of miR‐25‐3p inhibitor in CHB‐PNALT‐Exo (≥A2)‐treated HepG2.2.15 cells (Figure 9F).

## 4 DISCUSSION

HBV causes a chronic liver infection (called CHB), which is common worldwide. CHB is one of the major risk factors for end‐stage liver diseases, such as liver cirrhosis and liver cancer.^18^ Present guidelines recommend anti‐viral treatment for CHB patients with ALT level > 2 times the ULN.^10^ CHB can easily be missed in patients with ALT levels, twice than that of ULN, which can later develop into cirrhosis of the liver or liver cancer. The liver inflammation grade of some CHB patients with PNALT is greater than or equal to A2, and these patients are more likely to develop cirrhosis of the liver or liver cancer. Therefore, the molecular mechanism underlying the development of liver cancer in CHB patients with PNALT and liver tissue inflammation grade ≥ A2 needs to be further studied. Exosomes have been shown to play important roles in the occurrence and development of numerous diseases.^19^, ^20^, ^21^ Our results showed that miR‐25‐3p levels were lower in exosomes secreted by CHB‐PNALT (≥A2 or <A2) patients than in exosomes secreted by HBV^+^ patients with liver cancer. However, miR‐25‐3p expression in exosomes secreted by CHB‐PNALT (≥A2) was higher than that in exosomes secreted by CHB‐PNALT (<A2) patients. Intriguingly, miR‐25‐3p expression was closely related to the overall survival rate (Table 2 and 4). Additionally, CHB‐PNALT‐Exo (≥A2) significantly promoted the proliferation and metastasis of HepG2.2.15 cells and CHB‐PNALT‐Exo (<A2) significantly inhibited the proliferation and metastasis of HepG2.2.15 cells (Figure 1). The development of liver cancer in CHB patients with PNALT and liver inflammation grade ≥ A2 was closely related to its exosomes. Furthermore, we found that overexpression of miR‐25‐3p significantly promoted the proliferation and metastasis of HepG2.2.15 cells, whereas knockdown of miR‐25‐3p significantly inhibited the proliferation and metastasis of HepG2.2.15 cells (Figure 2). These results indicated that the effects of exosomes secreted by CHB‐PNALT (≥A2) patients on cell viability, apoptosis, proliferation, invasion and migration were consistent with the effects of overexpression of miR‐25‐3p on cell viability, apoptosis, proliferation, invasion and migration. Therefore, we speculated that the liver cancer‐promoting effects of exosomes secreted by CHB‐PNALT (≥A2) patients are closely related to the upregulation of miR‐25‐3p. Exosomes secreted by CHB‐PNALT (≥A2) patients transfected with FAM‐labelled miR‐25‐3p inhibitor and labelled with Dil were incubated with HepG2.2.15 cells and assessed for cell viability, apoptosis, proliferation, invasion and migration. The results showed that knockdown of miR‐25‐3p reversed the cell viability‐, proliferation‐, invasion‐ and migration‐promoting and apoptosis‐inhibiting functions of exosomes secreted by CHB‐PNALT (≥A2) (Figure 3). Additionally, an in vivo experiment showed that the oncogenicity of exosomes secreted by CHB‐PNALT (≥A2) patients was inhibited by transfection of miR‐25‐3p inhibitors, and this knockdown of miR‐25‐3p decreased Ki67 expression and increased cleaved caspase‐3/‐9 expression in HepG2.2.15 cells treated with exosomes secreted by CHB‐PNALT (≥A2) patients (Figure 4). These results confirmed that exosomes secreted by CHB‐PNALT (≥A2) patients promoted the development of liver cancer by increasing miR‐25‐3p levels.

It is well known that miRNAs regulate the biological functions of tumour cells by acting on target genes. Bioinformatics analysis identified 14 overlapping genes using EVmiRNA, TargetScan, TCGA database and GSE701728 data set. The PPI network was constructed using STRING database. The results showed that the PPI network included transcription factor 21 (TCF21), hedgehog‐interacting protein (HHIP), contacting 4 (CNTN4), synaptotagmin 1 (SYT1), cyclin‐dependent kinase 5 regulatory subunit 1 (CDK5R1), centromere protein F (CENPF) and enhancer of zeste homolog 2 (E2H2). However, the SYT1 expression data were inconsistent in TCGA database, UALCAN database and GSE101728 data set, and CDK5R1 and CENPF expression levels were upregulated in TCGA database, UALCAN database and GSE101728 data set. In addition, TCF21 and HHIP were mainly enriched in “tube development,” whereas CNTN4 had no connection with “tube development” (Figure 5). Therefore, both TCF21 and HHIP, which were identified as target genes of miR‐25‐3p and were downregulated in liver cancer, were used to follow up studies (Figure 6). The results showed that TCF21 directly interacted with HHIP and was positively correlated with HHIP in CHB patients with PNALT and HBV‐positive patients with liver cancer (Figure 7). *TCF21*, which is located on chromosome 6q23‐q24, encodes a member of the basic helix‐loop‐helix (bHLH) TF family.^22^ TCF21 plays a critical role during embryogenesis and in the development of numerous cell types in the heart, lung, kidney and liver.^23^, ^24^ TCF21 functions as an anti‐oncogene and can inhibit tumour cell proliferation and metastasis, and vascular production in breast cancer,^25^ ovarian cancer,^26^ lung cancer^27^ and liver cancer.^24^ HHIP is also an anti‐tumour gene and a negative feedback factor in the HH pathway that directly inhibits HH. HHIP, which is encoded by a gene located at 4q31.21‐31.3, can compete with PTCH for binding to hedgehog (Hh) protein, thereby blocking HH signalling.^28^ Studies have shown that while HHIP mRNA is expressed in normal tissues, its expression is decreased in some tumour tissues.^29^ It has been reported that overexpression of HHIP inhibited tumour cell proliferation and metastasis of lung cancer^28^ and gastric cancer.^30^ However, the related mechanisms and the role of HHIP in liver cancer have not been reported. Our results showed that inhibition of TCF21 and inhibition of HHIP promoted the proliferation, invasion and migration, and inhibited the apoptosis in HepG2.2.15 cells. However, the cell proliferation‐, invasion‐, migration‐ and apoptosis‐regulating effects of HHIP siRNA were counteracted by overexpression of TCF21 (Figure 8). This shows that TCF21 and HHIP have synergistic effects on the viability, proliferation, apoptosis, invasion and migration of HepG2.2.15 cells and both TCF21 and HHIP play extremely important anti‐tumour roles in HBV‐positive liver cancer cells. Furthermore, the functions of miR‐25‐3p inhibitors that mediated blockage of the effects of CHB‐PNALT‐Exo (≥A2) on the proliferation and metastasis were reversed by knockdown of TCF21 and HHIP in HepG2.2.15 cells (Figure 9). Ki67, caspase‐3/‐9 and E‐cadherin are marker proteins related to proliferation, apoptosis and metastasis.^31^, ^32^, ^33^ Our results showed that knockdown of TCF21 and HHIP abolished the cleaved caspase‐3/‐9, Ki67 and E‐cadherin expression‐regulating effects of the miR‐25‐3p inhibitor in CHB‐PNALT‐Exo (≥A2)‐treated HepG2.2.15 cells (Figure 9). These results indicated that transfer of miR‐25‐3p‐containing CHB‐PNALT‐Exo promoted the proliferation and metastasis of HBV‐positive liver cancer by inhibiting the co‐expression of TCF21 and HHIP.

## 5 CONCLUSIONS

The expression of miR‐25‐3p was upregulated in exosomes secreted by CHB patients with PNALT and liver tissue inflammation grade ≥ A2 and was closely related to poor survival in HBV‐positive patients with liver cancer. Exosomes derived from CHB patients with PNALT and liver tissue inflammation grade ≥ A2 promoted the occurrence and development of HBV‐positive liver cancer, and these effects were reversed by inhibition of miR‐25‐3p. *TCF21* and *HHIP* are two target genes of miR‐25‐3p that have anti‐tumour effects in HBV‐positive liver cancer. Exosomes secreted by CHB patients with PNALT and liver tissue inflammation grade ≥ A2 promoted the development of liver cancer by transferring miR‐25‐3p to inhibit the co‐expression of TCF21 and HHIP.

## CONFLICT OF INTEREST

The authors declare that they have no competing interests.

## AUTHOR CONTRIBUTIONS

Yi Ouyang and Yujing Tang wrote the main manuscript. Yi Ouyang, Yujing Tang and Shifang Peng analysed the data. Lei Fu, Shifang Peng, Yi Ouyang and Wanfeng Wu performed the experiments. Deming Tan, Lei Fu and Xiaoyu Fu designed the study. All authors read and approved the final manuscript.

## ETHICAL APPROVAL

The use of human clinical tissues was approved by the Institutional Human Experiment and Ethics Committee of the Xiangya Hospital Affiliated to Central South University. All experiments were conducted under the rule of the Declaration of Helsinki. All animal experiments were approved by the Animal Care and Use Committee at Central South University.

## Data Availability

The data that support the findings of this study are available from the corresponding author upon reasonable request.
